# The Role of Nurses and the Facilitators and Barriers in Diabetes Care: A Mixed Methods Systematic Literature Review

**DOI:** 10.3390/bs9060061

**Published:** 2019-06-14

**Authors:** Monica Nikitara, Costas S Constantinou, Eleni Andreou, Marianna Diomidous

**Affiliations:** 1 School of Sciences and Engineering, Department of Life and Health Sciences, University of Nicosia, Nicosia 2417, Cyprus; andreou.el@unic.ac.cy; 2 Medical School, University of Nicosia, Nicosia 2417, Cyprus; constantinou.c@unic.ac.cy; 3 Department of Public Health, National and Kapodistrian University of Athens, 11527 Athens, Greece; mdiomidi@nurs.uoa.gr

**Keywords:** nurses, roles, diabetes, care, inpatient, facilitator, barriers

## Abstract

Background: The aim of this review is to identify the roles and activities of nurses working with people with diabetes and to examine the facilitators and barriers in caring for such people. Methods: A systematic review was conducted. From 531 abstracts reviewed, 29 studies were included (18 studies comprised questionnaire surveys, one was an intervention study, two used both questionnaires and interviews, and eight of them used interviews). Barriers and facilitators were extracted and combined using qualitative synthesis. Results: The literature review revealed three major roles and a number of barriers. A model for achieving enhanced nursing care of patients with diabetes has been developed according to the findings of this literature. Specifically, a stepladder suggesting that through better nursing training and education and by providing adequate resources, time, and synergies to diabetes specialists, nurses will be able to correctly perform their diabetes care roles, which include patient education, advanced care, and psychological support. Conclusions: Taking into serious consideration that a large number of hospital users are people with diabetes and that there is an inconsistency among countries about the work settings of Diabetes Specialist Nurses (DSNs), it is important to give greater focus to inpatient care and perhaps to enhance nurses’ roles by eliminating any barriers that prevent them from providing adequate quality care. Furthermore, integrated care involving the role of DSNs within the inpatient care would have been more beneficial for patients.

## 1. Introduction

Diabetes has become an increasingly serious health issue on a global scale with the number of people living with diabetes rising significantly over the last 35 years. According to the International Diabetes Federation, in 2017, approximately 425 million adults (20–79 years) were living with diabetes, and by 2045 this will rise to 629 million [1]. The report also shows that over the past decade the prevalence of diabetes has risen faster in low- and middle-income countries than in high-income countries. Adults who are diagnosed with diabetes have a 3.5 times higher risk of being hospitalized than are those without a history of diabetes, while those with prediabetes are 1.3 times more likely to be hospitalized [2]. Furthermore, national data in the UK confirms that on average a patient with diabetes spends longer in hospital than a patient without diabetes despite being admitted for the same procedure or condition other than diabetes [3]. Direct medical costs associated with diabetes include expenditures for preventing and treating diabetes and its complications and cover outpatient and emergency care, inpatient hospital care, and long-term care. The major diabetes cost drivers are hospital inpatient and outpatient care [4]. However, according to the NHS Institute for Innovation Improvement [3], less emphasis has been given to how the British NHS can best treat and support people with diabetes when they are admitted to hospital, particularly when the main reason for their admission is not related to their diabetes.

Despite the fact that diabetes is a global issue, there is no universal approach to treating patients. For example, there are countries where the health care system allows nurses to have a major role in treating and educating people with diabetes, whereas in other countries doctors have a dominant role in diabetes care. In response to the need for enhanced support of patients with diabetes, multiple changes have occurred in treatment and care of diabetic patients and nurses’ role, which aimed to face the increasing rate of diabetes morbidity. Such changes include the establishment of the position of Diabetes Specialist Nurse (DSN), which allows nurses to prescribe medicines in countries like the UK and to be involved in the various levels of the health care system and to not be confined to hospitals [5,6]. This development has been found to improve clinical outcomes, to reduce inappropriate referrals to secondary care, and to reduce outpatient attendances [7]. However, it is important to report that, although many countries adjusted the Diabetes Specialist Nurse to their health care systems, nurses’ roles and work settings differed among countries. For example, in Sweden and the Netherlands, more than half of DSNs work in integrated or community settings and have prescribing rights. In contrast, most DNS in Ireland are hospital-based and not all of them are allowed to prescribe [7]. However, there is strong evidence in the literature to show that nurses have a major effect when counselling patients on self-management of their disease, particularly when combined with the proactive care management model [8,9,10,11] and decision-making support [12].

Given the increased number of people with diabetes in hospital wards and the developments described above, it would be reasonable to presume that the majority of nurses would have adequate experience and knowledge about diabetes inpatient care, and that patients with diabetes would receive adequate and high-quality care. Interestingly, there is evidence in the literature from past decades that patients frequently reported poor experiences of inpatient care particularly in relation to the lack of diabetes knowledge among hospital staff, especially nurses [13,14,15], inadequate information, and delays in being discharged resulting from diabetes, particularly when diabetes was not the original reason for admission [16]. Furthermore, no studies could be found in the literature about any internationally or even nationally agreed roles or responsibilities for diabetes for non-specialized nurses.

Therefore, in order to gauge the current situation of diabetes care, to explore any problems, and to improve the situation of diabetes care that nurses provide, there is a need to understand their roles in diabetes care and what facilitators and barriers nurses have when performing these roles. This mixed methods review of the literature aims to identify the roles and activities of the nurses working with people with diabetes and to examine the facilitators and barriers in caring for people with diabetes. The sample in this review included all the nurses that deal with diabetes care in every setting in order to understand the general situation and identify any gaps. Furthermore, this study focuses on both diabetes type 1 and type 2 since our aim is to identify what is the situation in general about diabetes care and whether there is a gap between these two types. To address this aim, the following search strategy and methodology were employed.

## 2. Methods

### 2.1. Type of Review

This review used both qualitative and quantitative studies, and it is therefore classified as a mixed methods review in accordance to the Joanna Briggs Institute’s definition. The mixed methods review allows for examining complex questions at different levels and combining findings investigated in different ways. In order to ensure the reliability and validity of the mixed methods review, we followed the guidelines of Joanna Briggs model for mixed methods review [17]. We formulated specific research questions in order to focus the review’s search strategy and sampling. 

### 2.2. Research Questions

The research questions of this review were as follows:

- What are the roles and activities of nurses caring for people with diabetes?
- What is the level of knowledge of nurses about diabetes care?
- What are the facilitators or barriers in caring for people with diabetes and in educating them about self-care management?

### 2.3. Search Strategy

A comprehensive and systematic review was conducted using the guidelines set forth in the Preferred Reporting Items for Systematic Reviews and Meta-Analyses (PRISMA) statement [18]. A search was conducted using CINAHL, MEDLINE, and Health Source, which are nursing/academic edition databases, for the period from January 1999 to January 2018. Keywords included, alone or in combination: “nurses”, “roles”, “activities”, “diabetes”, “care”, “barriers”, “facilitators”, and “inpatient”.

However, many of these studies were excluded because they referred to diseases other than diabetes, such as stroke, heart, or kidney failure. All articles were reviewed independently by two researchers who screened the titles and the subsequent abstracts separately, based on the inclusion criteria. Only primary research papers that reported nurses’ roles and knowledge and the facilitators or barriers to diabetes management were included. 

In summary, the articles had to meet the following inclusion criteria:

- To be focused on diabetes care;
- To articulate the roles of nurses or their knowledge;
- To include nurses in the study sample;
- To be written in English or Greek;
- To be published between 1999 and 2018;
- To be primary sources.

The studies included in this review are of a qualitative or quantitative nature, and concern nurses providing diabetes care who may be working in any setting.

### 2.4. Critical Appraisal

All studies that met the inclusion criteria were evaluated by two independent reviewers on their quality using the Joanna Briggs Institute Qualitative Assessment and Review Instrument Critical Appraisal Checklist for Studies Reporting Prevalence Data- Results, for Randomized Controlled Trials-Results, for Qualitative Studies-Results, for Case Series and for Analytical Cross Sectional Studies [17] (Appendix A).

### 2.5. Data Extraction

The extracted data included authors, title, year of publication, methodology aims/purposes, sample, instruments, and findings. The data extraction was implemented by two researchers and was checked for accuracy by a third researcher. 

### 2.6. Data Synthesis

Content analysis was used to synthesize the data. It is a method that involved analyzing the text of stories for their implicit meanings [19], and specifically, it refers to the systematic means of categorizing the findings into themes. During this review, content analysis was used to categorize the roles nurses cited they performed in inpatient diabetes care and the facilitators or barriers to fulfilling them, following a process proposed by Zhang and Wildemuth [20].

## 3. Results

**Figure 1:**
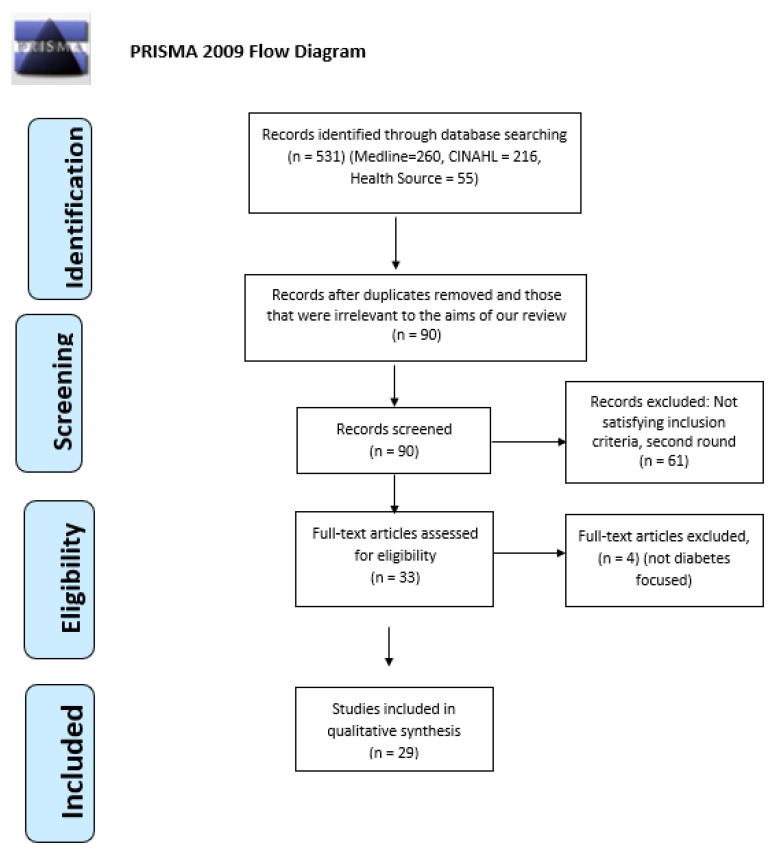
PRISMA flowchart with the search strategy of the systematic review.

The search procedure yielded 531 articles. All of the titles were checked and duplicates were excluded as well as those that were irrelevant to the aims of the review. A few of them referred not to diabetes care but to other diseases, or they were not specific to any disease. Some were not primary resources, and therefore, they were excluded (=441). The full texts of all abstracts (n = 90) were read to assess their relevance to the research topic and if they met the inclusion criteria. A total of 29 articles met the inclusion criteria and were included in this review (Figure 1). This review identified one important gap, which is the insufficient number of studies specifically regarding the role of hospital-based nurses in inpatient diabetes care and their perceptions about barriers to effective diabetes management.

The studies reviewed used a mixture of quantitative and qualitative methods or both methods: 19 studies comprised questionnaire surveys, two used both questionnaires and interviews, and eight of them used interviews. All of them are listed in Table 1. Studies have used either existing questionnaires or created new questionnaires to reflect current standards about diabetes. 

### 3.1. Nurses’ Roles in Diabetes Care

Various nursing roles emerged from the review, including nurses as educators, nurses as advanced caregivers, and nurses as motivators. Each of these is discussed below.

#### 3.1.1. Nurses as Educators

Various studies have explored the topic of nursing care and have found that nurses are greatly involved in educating patients to manage their disease [21,22,23], and some studies show the changing role of nurses in diabetes education [24]. Further studies have shown the positive outcomes that education has on patient condition when nurses are involved and the importance of diabetes education in improving glycemic controls [23,25]. More specifically, in a recent descriptive exploratory study, Bostrom et al. [21] showed the importance of a nurse’s role in patient education, with the Diabetes Specialist Nurse (DSNs) participants claiming that one of their roles was “being the teacher”, and they described how they educated patients about their new situation, informing them about the disease, possible complications, and test results.

The importance of diabetes education is also highlighted and supported by two studies. Wexler et al. [23] showed this through a randomized trial study with two groups. In their study, one group received normal care, and the other received both intervention care and formal education from experts such as specialist nurses who were approved at the beginning of the study. The findings of the study showed that the mean glucose levels were lower for the inpatients in the intervention group than in the usual care group. In the year after discharge, the average glycosylated hemoglobin (HbA1c) reduction was greater in the intervention group. The HbA1c test is an important measurement that shows the average blood glucose levels in the last three months. Similarly, Raballo et al.’s [25] study, in which patients received either usual care or group care, found more positive outcomes for those in group care. Their results show mostly positive attitudes in the patients that were assigned to group care, in contrast to those who had traditional visits. Additionally, patients in group care expressed a wider, more articulated range of concepts associated with the care they received than those who received usual care and who mostly expressed concepts with negative connotations. In general, the results suggest that patients under usual care tend to describe their condition and setting of care with concepts that mostly imply negative attitudes, poor empowerment, and an external locus of control. 

These studies indicated the changing role of the nurses in diabetes education and the importance of diabetes education in improving glycemic controls. Also, it is obvious from the above studies that nurses generally have the responsibility to educate diabetic patients.

#### 3.1.2. Nurses as Advanced Caregivers

Under the UK regulatory board and the Nursing and Midwifery Council (NMC) (2005) definition for APNs (Advance Practice Nurses) [26], skilled nurses could conduct several roles, such as deciding on and carrying out treatment, carrying out physical examinations, being a team leader, and making sure that each patient’s treatment and care is based on best practice, etc. [27]. The literature is replete with research describing nurses’ advanced role when caring for diabetic patients.

One element of advanced care found in the current literature is that advance nurse practitioners are involved in the management of medicines specifically for patients with diabetes, [28] which is a role that unspecialized nurses usually perform when they are caring for hospitalized patients. Furthermore, numerous studies referred to the nurses’ roles in prescribing medication, with some of them showing differences as to the extent to which nurses have that responsibility. For example, Carey and Courtenay [29] found that over two-thirds of respondents who are specialized nurses in the UK prescribed medications for common complications of diabetes, including hypertension, hyperlipidemia, and cardiovascular disease, although they devoted less than 20% of their week to doing that. This shows that the majority of their time nurses deal with other nursing care activities, and only a minimum time is devoted to advising on and ordering medication. However, there are no specific indications or guidelines in regards to how much of their time nurses have to devote to writing prescriptions. This is supported by earlier studies [24] which found that nurses’ contribution was limited to adjusting medication the patients took, since only 17.5% of nurses expressed their involvement in this in 1990 and fewer of them, 15.6%, in 1999 (x^2^ = 0.105, d.f. 1, *p* = 0.75). Also, this is supported by a more recent study which found that, although 77% of trusts in the UK had one or more nurse who had attended a nurse prescribing course, nurses did that in only 48% of responding trusts [22]. Therefore, it is obvious that, despite the fact that nurses through the years are receiving more advance knowledge and skills for the management of medications for people with diabetes, there are still concerns with regards to their actual engagement in that role.

The literature also highlighted the importance of nurses screening for diabetes complications, which is also within the role of an advanced practice nurse. Studies showed that nurses are involved in screening for complications in eyes and feet [30], whereas other studies referred to the role of the nurses to brief doctors about complications or problems [31].

There is evidence to suggest that nurses are more involved in conducting administrative duties for diabetes care through their daily routine. Specifically, two studies referring to the tasks that nurses have included managerial responsibilities [31] and that nurses are “striving to be an executive”, “being the bureaucrat”, and “being the administrator” [21].

Another role of nurses that was found in the literature review is that of collaborator. Fulfilling the role as a doctor’s assistant is an essential feature of the profession, and nurses believed that getting the work done, such as helping the doctor rather than providing effective care, was more important [32]. In other words, nurses preferred to conduct activities as per the physicians’ orders instead of spending time with patients to educate and support them. Furthermore, evidence was found that General Practitioners (GPs) often acted on practice nurses’ assessments of patients, and this shows that GPs trust PNs’ assessments [30] and that nurses thought they acted as intermediaries between doctor and patient, briefed doctors about complications or problems, helped doctors in treatment recommendations, and advised doctors about medications [31]. Furthermore, nurses organized and planned diabetes care between themselves, physicians, and other professionals, while they emphasized that they shared their mission in diabetes care with other professionals [21].

#### 3.1.3. Nurses as Motivators

The literature also showed that nurses undertake the role of motivator to diabetic patients. Several studies [28,31,33] have shown the importance of nurses in diabetic patients’ psychological support. Peyrot et al. [33] reported that nurses in comparison with doctors perceive greater needs in patients and see psychosocial problems as having great impact on self-care and control of patients with diabetes. Also, even though nurses provide more psychosocial care, they see themselves as less able to take care of a patient’s psychosocial needs in comparison to taking care of their physical needs. They also report great availability of psychosocial specialists and more often refer patients to them. Similarly, another study [31] found that nurses perceive it to be important to help their diabetes patients feel secure and hopeful. This is further supported by a study [28] which found that nurses participated in helping patients to address denial and illiteracy. This is further supported by another study [34] which found four strategies that nurses used to encourage patients: Educating for empowerment, advocating and reflecting on actions, treasuring the relationship, and humanizing complexity. 

### 3.2. Barriers or Facilitators for Nurses to Diabetes Care

Even though nurses now have many roles to perform, studies revealed that some barriers are preventing nurses from performing these roles, and only one facilitator has been identified in the literature that allows them to perform such roles.

#### 3.2.1. Nurses’ Lack of Knowledge 

Several previous studies revealed that nurses lack knowledge in specific areas of diabetes. This finding is further supported by recent literature that showed that nurses had poor understanding of more practical aspects of diabetes care such as knowledge about the timing and administration of some insulins, the use of metformin in renal impairment, when to escalate blood ketone results [35], and how to manage and recognize hypoglycemia symptoms [36,37]. Furthermore, it has been found that nurses are uninformed regarding insulin treatment despite their active involvement in the medical management of diabetic people [13,38]. Additionally, there is evidence suggesting that nurses do not have training regarding foot care and preventing complications [39]. They are aware of foot care in general, but they were unaware of other long-term complications of diabetes and lack of up-to-date information [14,40].

Recent studies show that nurses have deficient knowledge because they use outdated information about diabetes [41]. Their education about diabetes management was limited [30], and they have contradictory information because they had used obsolete and inaccurate textbooks when they were students preparing to be licensed nurses [13]. Furthermore, there is evidence suggesting that inadequate knowledge might come either from a deficit in the level of education in nursing school or that the nurses have not retained what they had been taught [30,42,43]. All of these studies referred to general nurses and not to specialized nurses. No studies were found in the literature regarding the level of knowledge of DSNs, and the lack of research on this is considered reasonable since DSN is a specialization and the nurses who acquired this title underwent a specific program in order to acquire expertise in the field of diabetes.

#### 3.2.2. Lack of Resources

There is some evidence supporting that one of the barriers nurses have to diabetes care is the lack of resources. An exploratory study investigated school nurses’ perceptions of self-efficacy and the factors related to self-efficacy in caring for and educating students on diabetes [44]. The findings indicate that nurses reported lower confidence in fulfilling specific tasks such as educating patients about diabetes when they do not have access to resources like measuring equipment. According to the authors, the lack of structured resources affects nurses’ confidence to give diabetes education in schools. Another study [45] showed that nurses perceived the main barrier to diabetes care was in relation to community resources and specifically to the lack of professional awareness of lifestyle programs and diabetes prevention initiatives for patients. This is further supported by two more studies [46,47]. O’Connor et al. [46] identified that the barriers nurses encountered to implementing diabetes care were the lack of equipment, lack of space, and lack of IT. In a study by Roopnarinesingh et al. [47] in which the majority of the participants were health professionals including nurses, they identified that the limitation in available resources was a significant barrier to diabetes care. The majority of participants reported that resources such as access to on-site blood testing, skilled ophthalmological evaluation and care, consultations for difficult to manage cases, on-site ECGs, and cardiac stress testing were inadequate or not being optimally utilized. 

#### 3.2.3. Lack of Time 

One of the most important barriers in caring for diabetes patients is nurses’ lack of time. Nurses indicated that time constraints were one of the biggest challenges in providing support and effective diabetes management [30,46,48]. Joshi et al. [48] investigated school nurses’ perceptions of what inhibited them in teaching patients about diabetes and its management and whether using technology can overcome such barriers. The results of the study show that the nurse participants indicated that time constraints were one of the biggest challenges in providing support and care to children with diabetes. The lack of access to diabetes education was another major problem they encountered. These barriers are important since they seriously impaired the nurses’ ability to provide adequate care. These findings are in line with Livingston and Dunning’s work, in which results showed nurses believed they lack sufficient time to provide effective diabetes management [30]. This is remarkable since nurses identified patient education as one of their most important activities they carry out, but they do not do so because of insufficient time. Furthermore, another study also found that nurses said having inadequate time to screen for complications was an important barrier to giving optimal care [47]. The majority of participants reported that there was neither sufficient time to screen and evaluate diabetic complications nor time and resources to routinely evaluate for heart and vascular complications. O’Connor et al. [46] also reported that all health care professionals including nurses noted time constraints as a barrier to diabetes care and that the demands on their time were ever increasing, with many nurses stretched to the limit.

### 3.3. Collaboration with Diabetes Specialists

Some studies showed that when nurses collaborate with diabetes specialists their involvement with patient education is low, and this is the only finding in this review that could be perceived as either a facilitator or a barrier to diabetes care. For example, some participants reported that nurses are not actively involved in teaching patients with diabetes, but other participants thought they relied on educators to teach patients with diabetes and did not attempt to enhance their own knowledge [13,15]. On the other hand, multidisciplinary collaboration was found to be a major facilitator to diabetes care, although collaboration with certain professional groups such as dieticians, physical therapists, and pharmacists could be further improved as could teamwork between primary and secondary caregivers [45]. 

## 4. Discussion

It is clear from this systematic review of the literature that the complex nature of diabetes necessitates the involvement of nurses who have multiple roles in providing care in order to achieve effective management of the disease. Despite the fact that health care systems in many countries incorporate the role of the DSNs in diabetes care, usually the nurses who provide diabetes care at the bedside are nurses without any specialization in diabetes. This systematic literature review revealed the following three major roles: Nurses as educators, nurses as advanced caregivers, and nurses as motivators. Interestingly, despite the fact that adults who are diagnosed with diabetes have a 3.5 increased chance of being hospitalized than are those without a history of diabetes, no studies were found that solely refer to nursing roles in inpatient diabetes care, with most of the studies referring to the primary setting or to the roles of the Diabetes Specialist Nurse (DSN) and their effect on diabetes care. However, as has been previously said, the DSN role is a new development to face diabetes, but their roles and work settings differed between countries, with most of them working in primary care. Therefore, it is apparent that great emphasis has been given to primary care for diabetes. However, the fact that many hospitalized patients have a known diabetes history indicates the need for further research and focus on inpatient diabetes care and on how people with diabetes can best be treated and supported. 

Another important finding is that diabetes education is the cornerstone of diabetes management, and this systematic review showed that nurses with or without extra education in diabetes care mostly recognize that educating diabetic patients is something they must do. Several elements of nurses’ roles in diabetes care, as found in the review, adhered to the definition that the UK’s regulatory board, the Nursing and Midwifery Council (NMC) [26], has for Advanced Nurse Practices (APNs). These practices include administering and prescribing medication, screening for complications, and administration. Lastly, one of the important roles of nurses that the literature identified is to offer psychological support to diabetic patients. 

This systematic literature review also demonstrated that nurses and other health care professionals encounter only one facilitator, which is the collaboration with other diabetes specialists. On the other hand, they encounter various barriers to achieving optimal diabetes care. One of the most important findings regarding the barriers is nurses’ lack of knowledge about diabetes, which remains an obstacle to diabetes care. It is evident that nurses have concerns about their knowledge of diabetes care, and this supports the view that people with diabetes frequently reported poor experiences during inpatient care, particularly in relation to the lack of diabetes knowledge among hospital staff, especially nurses. Therefore, there still is a need to further train nurses about diabetes care in conjunction with the integration of DNS on inpatient care. Two other barriers that were frequently seen in the literature were lack of resources and lack of time. Only nurses’ collaboration with diabetes specialists was considered as both a facilitator and a barrier to diabetes care. 

**Figure 2:**
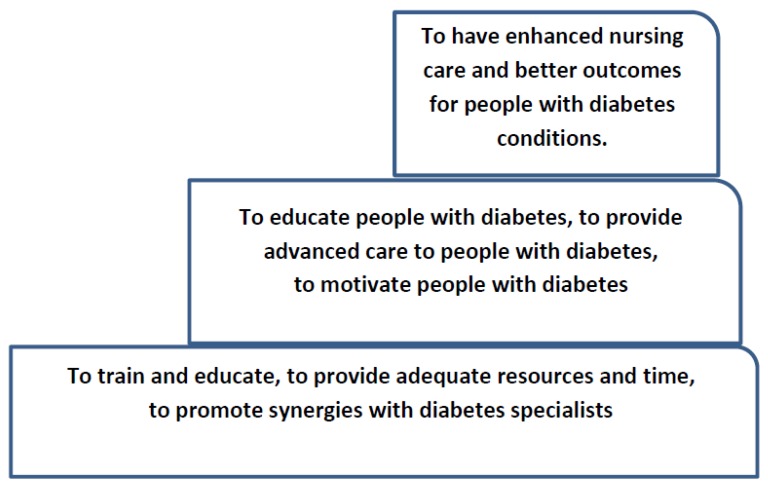
The stepladder model for achieving enhanced nursing care of people with diabetes.

Having identified the role of nurses in diabetes care and the barriers and facilitators they encounter in fulfilling their roles in the existing literature and having in mind the documented contribution of nurses to improving the health outcomes of patient with diabetes [23,25], we propose a model for achieving enhanced nursing care of patients with diabetes. More specifically, our model suggests a three-rung stepladder (Figure 2). To better explain the model, we will start from the third and final step, which is the desired outcome, that is, to enhance nursing care and to achieve better health outcomes. In order to approach the third step successfully, nurses have multiple roles to play such as to be patient educator, advanced caregiver, and patient motivator. These roles, however, cannot be fulfilled adequately if nurses are not well supported at the first and most basic step, which should include training, having appropriate resources and time, and collaborating with relevant specialists. This stepladder model could take the form of an evaluation program, and it could be tested quantitatively in a randomized control study. That is, a group of nurses could be trained and supported, assigned specific roles in diabetes care (the intervention group), and compared with another group of nurses with no specialized training or specific diabetes care roles. Both groups could then be compared with their patients’ health outcomes (e.g., diet, exercise, and glucose control). 

## 5. Limitations of the Study

First of all, the inconsistency between the different titles and specialties of the nurses included in the studies led to challenges in discovering articles to include in this study. Two of the articles included only school nurses and one used mental health nurses in its sample, and this is not representative of the whole population of nurses [41,44,48]. Furthermore, some of the studies did not report the response rate of the participants, and some of them reported a low response rate [13,14,15,40].

## 6. Conclusions

Despite the developments and initiatives that have taken place over the last years in order to confront the disease of diabetes, the statistics still highlight the large number of people with diabetes, which is increasing worldwide. Therefore, since nurses have an important role in being involved in diabetes care, it is of great importance to clearly identify their multiple and sometimes complicated roles in diabetes care, to eliminate any barriers that prevent them from providing adequate care, and to enhance any facilitators that allow them to provide the best quality care. Finally, this literature review showed the importance of assigning more diabetes-specific roles to nurses and supporting nurses in order to achieve positive health outcomes as per the stepladder model we propose.

## Figures and Tables

**Table 1:** Study characteristics.

N	Authors and Year	Methodology	Sample	Relevant Findings
**1**	Livingston, R. and Dunning, T. (2010)	Mixed	21 nurses	• Inadequate knowledge • Deficit in nurses’ knowledge (risk factors, treatment, complications) • Distance from education • Difficulties collaborating with diabetes specialist services • Lack of experience and age
**2**	Chan, M. F. and Zang, Y (2007)	Quantitative	245 nurses	**Cluster 1**: Highly competent and knowledgeable about diabetes (40.4%) **Cluster 2**: Moderately competent and knowledgeable (43%) **Cluster 3**: Low competence and low diabetes knowledge. (16.3%) Also fewer years of work experience and lower education level • Question on competence: more than 57% of all nurses reported somewhat/not very/not at all competent but there were differences between clusters–statistically significant differences found on the competence between the three Clusters (*p* < 0.001). • Perceived knowledge was significant and positively correlated with their actual knowledge (rs = 0.32, *p* < 0.001).
**3**	Lipman, T. H, and Mahon, M. M. (1999)	Quantitative	155: Nurses, nursing students, and non-nursing students	• Diabetes knowledge found to be lacking in all groups The mean scores were as follows: Group I = 65.3% (±14.4%); Group II = 57.4% (±17.3%); Group III = 13.1% (±11.0%). The scores for practicing RNs ranged from 20% to 95% (mean = 64.3% ±14.4%). • RNs’ knowledge was poorest regarding insulin action and in signs, symptoms and treatment of hyperglycemia. • Deficient knowledge either of diabetes education in nursing school or in the retention of what has been taught • Misinformation/ outdated textbooks • Student nurses’ poor knowledge of what glucagon is and when it should be used • Staff nurses are not motivated to learn about diabetes.
**4**	Fisher, K. L (2006)	Quantitative	70 school nurses	• Nurses perceived they were moderately confident regarding diabetes care and education for children—total score on Self-Efficacy on Diabetes Education (SEDE) (mean = 36.30, SD 9.99). • Respondents reported lower confidence scores for (a) educating about diabetes when they cannot access measuring equipment, (b) inaccessible resources (mean = 2.78, SD 1.09).
**5**	El-Deirawi, K.M and Zuraikat, N. (2001)	Quantitative	79 Nurses: 32 community hospital nurses, 47 home health care agency nurses	• Small but significant, positive correlation (r = 0.402, *p* < 0.0001) between perceived and actual knowledge of diabetes Participants’ lack of knowledge about insulin injections sites and etiology of type 1 diabetes mellitus • Home health nurses achieved higher scores than hospital nurses on the DSRT (Diabetes Self-report Tool) (t = 3.835, *p* < 0.0001). • The level of education was found to be positively correlated with the level of knowledge of diabetes. • The participants either attended not in-service education about diabetes or attended in-service programmes more than 2 years ago.
**6**	Speight, J. and Bradley C. (2001)	Quantitative	789 patients and 16 health professionals	• Knowledge scores were higher in insulin-treated than in non-insulin-treated patients (U = 34875.0; n = 422 and 323, respectively; *p* < 0.0001). • Knowledge of foot care, hypoglycemia, and diet and food were the most problematic topics, with patients achieving mean scores of 59.5%, 62.4%, and 64.5%, respectively, for these topics. • Patients and health professionals show deficit knowledge on items that might have serious short and long terms consequences.
**7**	Joshi, A., Komlodi, A. and Arora, M. (2008)	Quantitative	43 school nurses	• 79% use internet as the main information source • Barriers of communication between health care providers of the diabetic students • Providers lack access and time. • Time constraints and lack of access to diabetes education in acquiring diabetes knowledge • Lack of updated relevant clinical information
**8**	Derr R., Sivanandy M., Bronich-Hall L., and Rondriguez A. (2007)	Quantitative	377: 73 faculty, 113 residents, and 191 nurses	• The majority of general medicine faculty felt very comfortable in managing diabetes and the majority of other categories described themselves as somewhat comfortable. • For the 16 knowledge-based questions, the overall percentages answered correctly was 51% for faculty, 59% house staff and 47% for nurses. • Nurses scored worse than physicians on questions testing knowledge of inpatient insulin use (34% vs. 57%, *p* < 0.0001).
**9**	Nash M. (2009)	Quantitative	138 mental health nurses	• The majority of the sample self-rated their knowledge of diabetes knowledge as fair (40%). • 64% of the sample felt that they had not received appropriate training. • 86% indicated that they required further training.
**10**	Shiu, A. and Wong, R. (2011)	Quantitative	65 Registered nurses	• 57% claimed that they had never received any training in diabetes foot care knowledge. • The level of registered nurses’ foot care knowledge was fair. • RNs with (n = 22, mean DFKS = 41.5, SD 7.3) and without (n = 43, mean DFKS = 41.3, SD 6.1) work experience in diabetes care specialty services obtained similar DFKS marks, *p* = 0.924. (DFKS = Diabetes Foot Care Knowledge Scale).
**11**	Findlow, L. and McDowell, J. (2002)	Quantitative	133 registered nurses	• Only 5.2% attended a diabetes course within the preceding five years. • The overall pass rate was 69%. • Comparison of results by grade showed that there was no statistically significant difference in knowledge between the two groups of nurses (grades D/E and F/G, *p* = 0.255). (Grades are according to the nurses’ enrolment status). • Almost half of the respondents (48.4%) were unaware of how to carry out blood glucose monitoring accurately and precisely.
**12**	Wexller, D, Veauharnais, C., Regan, S., Nathan, D., Cagliero, E., and Larkin, M. E. (2012)	Quantitative	31 patients	• Mean inpatient glucose was lower in the IDMET than in the UC group (*p* = 0.001). • After one year of discharge, the average HbA1c reduction was greater in IDMET compared to the UC group (*p* = 0.5). • Newly discharged patients on insulin; the average HbA1c reduction was greater in the IDMET than in the UC group.
**13**	Peyrot, M., Rubin, R., and Simnierio, L. (2006)	Qualitative	2,705 physicians, 1,122 nurses	• Nurses perceived significantly higher prevalence and severity of psychosocial problems and used psychosocial strategies significantly more frequently than physicians. • Both physicians and nurses diabetes specialists were significantly more likely than generalists to utilize psychosocial strategies.
**14**	Mutea N. and Baker C. (2007)	Qualitative	15 registered nurses	Content analysis produced 8 categories of nurses’ involvement in managing diabetic patients: • Knowledge of DM • Management of DM • Continuity of care • Quality of care • Patient accessibility to diabetic care • Patient support activities • Resources • Condition of service
**15**	Kenealy, T., Arroll, B., Kenealy, H, Docherty, B., Scott, D., Scragg, R., and Simmons, D. (2004)	Quantitative	86 PNs in 1990 77 PNs in 1999	• In 1999, nurses looked after more patients with diabetes without spending more time on diabetes care than nurses in 1990. • Nurses in 1999 reported increased involvement in the more complex areas of diabetes care such as foot care (61% vs. 40% x^2^ = 7.339, d.f. 1, *p* = 0.021). • In 1999, nurses were no more likely than those in 1990 to adjust treatment. Proposals were debated to allow nurses to prescribe.
**16**	Kassean H. K. (2005)	Qualitative	10 nurses	Four main themes emerged: (1) Management of care (2) Barriers to care (3) Communication skills (4) Training and educational needs • The majority of nurses did not feel knowledgeable. • Deficit in their knowledge
**17**	James J., Gosden C., Winocour P., Walton C., Nagi D., Turner B., Williams R., and Holt RI. (2009)	Quantitative	159 (DSNs, Nurse consultants and diabetes health care assistants)	• 78% and 76% DSNs planned and delivered education sessions compared with 13% in 2000. • 22% of DSN had a formal role in diabetes research compared with 48% in 2000 (*p* <0.000). • 49% of hospital DSNs, 56% of Community DSNs, and 66% of nurse-consultants are involved in prescribing.
**18**	Carey, N. and Courtenay, M. (2008)	Quantitative	214 Nurses located throughout the UK	• The majority of nurses prescribe between one and five items a week–differences between groups were statistically significant (*p* = 0.001). • Oral anti-diabetic drugs, hypertension and lipid regulating drugs, and insulin were often prescribed. (*p* = 0.031).
**19**	Bostrom, E., Isaksson, U., Sjolander, A., and Hornsten, A. (2012)	Qualitative	29 DSNs	• DSNs identify five major roles of their profession: expert, fosterer, executive, leader, and role model. • Challenges interpreted as role ambiguities included feeling uninformed, fragmented, resigned, pressed for time, and self-reproachful.
**20**	Siminerio, L., Funnel, M., Peyrot, M. and Rubin, R. (2007)	Qualitative	51 generalist nurses, 50 specialist nurses, 166 generalist physicians, 50 diabetes specialist physicians	• Nurses and physicians agreed that nurses should take a larger role in managing diabetes. • Nurses provide better education, spend more time with patients, were better listeners, and knew their patients better than physicians. • All nurses had a high perceived need for better understanding of psychosocial issues. • Generalist nurses report that they act as intermediaries and facilitate patient appointment keeping.
**21**	Raaijmakers, L.G.M., Hamers, F.J.M., Martens, M. K., Bagchus, C., de Vries, N.K., and Kremers, S.P.J. (2013)	Qualitative	18 Health professionals: 3 Family Physicians, 3 Practice Nurses, 2 Diabetes Nurses, 3 Dieticians, 2 Physical Therapist, 3 Internal Medicine Physicians, 2 Pharmacists	• Major facilitators: More prominent role of practice nurses and diabetes nurses in diabetes care • Certain professional groups collaborate (i.e., dieticians, physical therapists, and pharmacists); there is collaboration between primary and secondary care, but could improve. • Health insurers’ bundled payment system for funding diabetes care was perceived as a major barrier within the health care system. • Patients’ lack of motivation and their lack of awareness of lifestyle programmes and prevention initiatives were barriers.
**22**	O’Connor, R., Mannix, M, Mullen, J, Powys, L, Mannion, M, Nolan, HA, Kearney, E, Cullen, W, Griffin, M, and Saunders, J. (2013)	Qualitative	GPs (n = 55) practice nurses (n = 11)	Distinct barriers and facilitators emerged in relation to the proposed change in structured diabetes care within general practice. They fell into three domains: practitioner factors, practice factors, and systemic factors.
**23**	Roopnarinesingh, N, Brennan, N., Khan, C., Ladenson, PW, and Hill-Briggs, F. (2015)	Quantitative	198 Participants physicians (44%, n = 88) or nurses (40%, n = 79)	Barriers reported: Limited access to blood testing (75%), ophthalmological evaluations (96%), ECGs (69%), and cardiac stress tests (92%); inadequate time to screen and evaluate DM complications (95%), poor access to consultants for referral of difficult cases (77%), and lack of provider education regarding cardiovascular complications of DM (57%). HCP agreed that nurses could have a more active role in the care and prevention of cardiovascular disease and diabetes through leading patient education efforts (98%), screening patients for complications (91%), coordinating care efforts (99%), and educating family members (98%).
**24**	Cardwell J., Hardy K., Ford N., and O’Brien S. (2016)	Quantitative	26 RGNs and 17 HCAs from general medical or surgical ward	• Both wards scored 100% on knowledge about physiology of diabetes, but they did not understand more practical elements. • This included knowledge about the timing and administration of some insulins, use of metformin in renal impairment, and when to escalate blood ketone results. • There was no significant difference in scores between the medical and surgical wards.
**25**	Chinnasamy E., Mandal A., Khan S., Iqbal F., and Patel N. (2011)	Quantitative	100 responses were received from participants: 80% staff nurses, 20% senior or charge nurses	• 51% had formal training in hypoglycemia management. • 28% nurses knew the common symptoms of hypoglycemia listed in the questionnaire. • 73% of the hypoglycemic episodes were detected by routine checks and this was predicted by nurses in the survey. Conclusion: There is a lack of knowledge among ward nurses regarding hypoglycemia management and further training is necessary.
**26**	Ndebu, J. and Jones, C. (2018)	Quantitative	40 responses received. The breakdown of staff was: 5% Ward Manager, 7.5% Senior Nurse; 75% Staff Nurse, 2.5% HCA (Band 4) and 10% HCA	93.75% (15/16) of diabetes ward nurses recognized all hypoglycemia symptoms. Only 58.3% (7/12) from vascular and 25% (3/12) from critical care wards recognized the symptoms. 58.3% (7/12) of critical care nursing staff recognized just 3 or fewer hypoglycemia symptoms. Although everyone used some form of rapid-acting carbohydrates for hypoglycemia treatment and were aware to recheck CBG after treatment, only 77.5% (31/40) of the nursing staff rechecked the CBG after 15 minutes as per this hospital’s guidelines, while 17.5% (7/40) rechecked after 20–30 minutes. Moreover, 10% (4/40) stated they would have omitted insulin after this treatment.
**27**	Modic, M. B., Vanderbilt, A., Siedlecki, S. L, Sauvey R., Kaser N., and Yager C., (2014)	Quantitative	2250 registered nurses working in a quaternary health care centre	• Nurses’ knowledge of inpatient diabetes management principles was low. No correlation between knowledge scores and age, education, employment status, years of experience, or clinical specialty. • Pearson’s correlation was conducted to determine if there was a relationship between comfort level or familiarity and diabetes management knowledge: there was no correlation between comfort (r = 0.002; *p* = 0.912) and familiarity (r = −0.013; *p* = 0.556) and diabetes management knowledge; correlation found between comfort and familiarity (r = 0.706; *p* < 0.001)
**28**	Raballo, M, Trevisan, M, Trinetta, A, Charrier, L, Cavallo, F, Porta, M, and Trento, M. (2012)	Mixed	241 patients	Patients on group care showed more positive attitudes, higher sense of empowerment, and more internal locus of control than those on usual care. In addition, they expressed a wider and more articulated range of concepts associated with the care received and made less use of medical terminology.
**29**	Donohue-Porter P. (2013)	Qualitative	14 nurses	• Educating for empowerment • Advocating and reflecting action • Treasuring the relationship • Humanizing the complexity

## Appendix A

**JBI Critical Appraisal Checklist for Studies Reporting Prevalence Data Results**

**Authors and Year**	**Q1**	**Q2**	**Q3**	**Q4**	**Q5**	**Q6**	**Q7**	**Q8**	**Q9**
Chan, M. F. and Zang, Y (2007)	√	√	√	√	√	√	√	√	√
Lipman, T. H, and Mahon, M. M. (1999)	√	√	√	√	√	√	√	√	No
Fisher, K. L (2006)	√	√	√	√	√	√	√	√	√
El-Deirawi, K.M and Zuraikat, N. (2001)	√	√	√	√	√	√	√	√	No
Speight, J. and Bradley C. (2001)	√	√	√	√	√	√	√	√	No
Joshi, A., Komlodi, A., and Arora, M. (2008)	√	√	√	√	√	√	√	√	√
Derr R., Sivanandy M., Bronich-Hall L., and Rondriguez A. (2007)	√	√	√	√	√	√	√	√	√
Nash M. (2009)	√	√	√	√	√	√	√	√	√
Shiu, A. and Wong, R. (2011)	√	√	√	√	√	√	√	√	√
Findlow, L. and McDowell, J. (2002)	√	√	√	√	√	√	√	√	No
Kenealy, T., Arroll, B., Kenealy, H, Docherty, B., Scott, D., Scragg, R., and Simmons, D. (2004)	√	√	√	√	√	√	√	√	√
James J., Gosden C., Winocour P., Walton C., Nagi D., Turner B., Williams R., and Holt RI. (2009)	√	√	√	√	√	√	√	√	√
Carey, N. and Courtenay, M. (2008)	√	√	√	√	√	√	√	√	√
Chinnasamy E., Mandal A., Khan S., Iqbal F., and Patel N. (2011)	√	√	√	√	√	√	√	√	√
Ndebu, J. and Jones, C. (2018)	√	√	√	√	√	√	√	√	√
Modic, M. B., Vanderbilt, A., Siedlecki, S. L, Sauvey R., Kaser N., and Yager C., (2014)	√	√	√	√	√	√	√	√	√
Roopnarinesingh, N, Brennan, N., Khan, C., Ladenson, PW, and Hill-Briggs, F. (2015)	√	√	√	√	√	√	√	√	√

**JBI Critical Appraisal Checklist for Randomized Controlled Trials Results**

**Authors and Year**	**Q1**	**Q2**	**Q3**	**Q4**	**Q5**	**Q6**	**Q7**	**Q8**	**Q9**	**Q10**	**Q11**	**Q12**	**Q13**
Wexller, D, Veauharnais, C., Regan, S., Nathan, D., Cagliero, E., and Larkin, M. E. (2012)	√	√	√	√	√	√	√	√	√	√	√	√	√
Raballo, M, Trevisan, M, Trinetta, A, Charrier, L, Cavallo, F, Porta, M, and Trento, M. (2012)	√	√	√	√	√	√	√	√	√	√	√	√	√

**Risk of Bias Assessed by the Joanna Briggs Institute Critical Appraisal Checklist for Qualitative Studies Results**

**Authors and Year**	**Q1**	**Q2**	**Q3**	**Q4**	**Q5**	**Q6**	**Q7**	**Q8**	**Q9**	**Q10**
Peyrot, M., Rubin, R., and Simnierio, L. (2006)	√	√	√	√	√	No	No	√	√	√
Mutea N. and Baker C. (2007)	√	√	√	√	√	No	No	√	√	√
Kassean H. K. (2005)	√	√	√	√	√	No	No	√	√	√
Bostrom, E., Isaksson, U., Sjolander, A., and Hornsten, A. (2012)	√	√	√	√	√	No	No	√	√	√
Siminerio, L., Funnel, M., Peyrot, M., and Rubin, R. (2007)	√	√	√	√	√	No	No	√	√	√
Raaijmakers, L.G.M., Hamers, F.J.M., Martens, M. K., Bagchus, C., de Vries, N.K., and Kremers, S.P.J. (2013)	√	√	√	√	√	No	No	√	√	√
O’Connor, R., Mannix, M, Mullen, J, Powys, L, Mannion, M, Nolan, HA, Kearney, E, Cullen, W, Griffin, M, and Saunders, J. (2013)	√	√	√	√	√	No	No	√	√	√
Donohue-Porter P. (2013)	√	√	√	√	√	√	√	√	√	√

**JBI Critical Appraisal Checklist for Case Series**

**Authors and Year**	**Q1**	**Q2**	**Q3**	**Q4**	**Q5**	**Q6**	**Q7**	**Q8**
Cardwell J., Hardy K., Ford N., and O’Brien S. (2016)	√	√	√	√	√	√	√	√

**JBI Critical Appraisal Checklist for Analytical Cross-Sectional Studies**

**Authors and Year**	**Q1**	**Q2**	**Q3**	**Q4**	**Q5**	**Q6**	**Q7**	**Q8**
Livingston, R. and Dunning, T. (2010)	√	√	√	√	√	√	√	√
